# A systematic review of the effects of acupuncture on xerostomia and hyposalivation

**DOI:** 10.1186/s12906-018-2124-x

**Published:** 2018-02-13

**Authors:** Zainab Assy, Henk S. Brand

**Affiliations:** Department of Oral Biochemistry, Academic Centre for Dentistry Amsterdam (ACTA), room 12N-37, Gustav Mahlerlaan 3004, 1081 LA Amsterdam, The Netherlands

**Keywords:** Acupuncture, Xerostomia, Hyposalivation, Salivary flow rate, Sjögren’s syndrome, Radiotherapy, Randomized controlled trials

## Abstract

**Background:**

Saliva is fundamental to our oral health and our well-being. Many factors can impair saliva secretion, such as adverse effects of prescribed medication, auto-immune diseases (for example Sjögren’s syndrome) and radiotherapy for head and neck cancers. Several studies have suggested a positive effect of acupuncture on oral dryness.

**Methods:**

Pubmed and Web of Science were electronically searched. Reference lists of the included studies and relevant reviews were manually searched. Studies that met the inclusion criteria were systematically evaluated. Two reviewers assessed each of the included studies to confirm eligibility and assessing the risk of bias.

**Results:**

Ten randomized controlled trials investigating the effect of acupuncture were included. Five trials compared acupuncture to sham/placebo acupuncture. Four trials compared acupuncture to oral hygiene/usual care. Only one clinical trial used oral care sessions as control group. For all the included studies, the quality for all the main outcomes has been assessed as low. Although some publications suggest a positive effect of acupuncture on either salivary flow rate or subjective dry mouth feeling, the studies are inconclusive about the potential effects of acupuncture.

**Conclusions:**

Insufficient evidence is available to conclude whether acupuncture is an evidence-based treatment option for xerostomia/hyposalivation. Further well-designed, larger, double blinded trials are required to determine the potential benefit of acupuncture. Sample size calculations should be performed before before initiating these studies.

## Background

Saliva is fundamental to our oral health and our well-being [1]. Important functions of saliva are lubrication, digestion, antibacterial/antifungal activity, buffering, remineralization, and production of growth factors and other regulatory peptides [2]. Furthermore, oral functions such as speaking, swallowing and tasting require saliva. When the protective function of saliva becomes insufficient, this has profound negative effects on the oral health. An impaired saliva secretion (hyposalivation) usually results in the feeling of a dry mouth (xerostomia). Other consequences are increased caries formation, increased rate of acute gingivitis, dysarthria, dysgeusia, increased rate of candidal infection and burning tongue [2]. Other negative effects are taste aberrations, breath malodor and poor denture retention [3, 4]. All these distressing symptoms have a profound negative impact on patients’ quality of life [5, 6].

Many factors can impair saliva secretion. The most frequent cause of xerostomia is use of medication. Especially anticholinergic medications (for example tricyclic antidepressants, antipsychotics) are notorious for their xerostomic side effects [7]. The risk of xerostomia increases with the number of medications being taken [8]. Another cause of xerostomia is radiotherapy and chemotherapy. Head and neck malignancies are treated with radiotherapy or chemotherapy or a combination of both. The severity of xerostomia is depending on the total exposure of the salivary glands to the radiation or the total number of chemotherapeutic drugs used [7]. Autoimmune disease such as Sjögren’s syndrome can also induce xerostomia. Other, less common causes of xerostomia include sarcoidosis, HIV disease and HCV infection.

Various therapeutic strategies are available for xerostomia. To apply the appropriate therapy, the residual secretory capacity of the salivary glands must be assessed. When the salivary glands still can be stimulated, gustatory and/or mechanical stimuli (mint flavoured sucking tablet or sugar free chewing gum) are useful [9]. If these stimuli are not effective, systemic administration of a cholinergic agonist can be considered. A well-known cholinergic agonist is pilocarpine. Pilocarpine can stimulate salivary flow in normal volunteers as well as in patients with xerostomia [2]. However, pilocarpine may have adverse side effects such as nausea, vomiting, increased urinary frequency and headache [10].

When stimulated saliva secretion is much reduced or when stimulation of saliva secretion is impossible, palliative oral care can alleviate xerostomia [9]. Widely used palliative oral care products include mouthwashes, oral gels and saliva substitutes. These products are available over the counter without prescription, and these products can reduce xerostomia symptoms, which in turn may improve the quality of life [1]. However, palliative care products have several limitations. They are removed from the mouth during swallowing, the duration of their effect is short and they also lack the protective effects of saliva [7]. Due to the limitations of the therapies described above, complementary and alternative medicine (CAM) have become more popular among patients suffering from xerostomia [11]. One of the most widely used CAM therapies is acupuncture.

Acupuncture means to ‘to puncture with a needle’ [12]. Acupuncture treatment involves the insertion of extremely thin solid needles into intradermal or subdermal loci for the therapeutic relief of many symptoms [13]. In 2003, the World Health Organization published a report on the efficacy of acupuncture in the cure or relief of 64 different symptoms [14].

There are several hypotheses how acupuncture can increase the salivary secretion. Acupuncture can stimulate the parasympathetic and sympathetic nervous systems by neuronal activations [12, 15]. Additionally, acupuncture therapy produces the release of neuropeptides such as the vasodilator calcitonin gene-related peptide [12, 15]. These neuropeptides have anti-inflammatory properties and trophic effects on the salivary gland and increase the blood flow in the acini [12]. Another explanation is that acupuncture can directly affect the local blood flow in the proximity of the salivary gland and thereby increase the salivary secretion [16]. Finally, acupuncture therapy may tap into the neuronal circuit, which activates the salivary nuclei in the pons and subsequently the salivary glands via the cranial nerves [12]. Acupuncture is a low risk therapy [17–20] and significant adverse events of acupuncture are rare (less than 1 per 20,000 individuals) [13].

Several studies have explored the effect of acupuncture on oral dryness [21, 22]. Although some of these studies suggest a positive effect, a systematic review of the effects of acupuncture on salivary secretion or xerostomia symptoms is still lacking. Therefore, the aim of the present is to investigate whether acupuncture is an evidence-based option for the treatment of xerostomia/hyposalivation, and - if this is the case - to ascertain which patients with oral dryness benefit from acupuncture.

## Methods

Systematic review of the literature was performed using the databases of Medline/Pubmed and Web of Science till July 2015. The electronic database of Pubmed was searched for articles using keywords related to acupuncture and xerostomia or hyposalivation. An initial search was conducted using the terms (salivary gland diseases) OR (salivary gland disease) OR (salivary glands) OR (“saliva”[MeSH Terms]) OR (“salivation”[MeSH Terms]) OR (saliva secretion) OR (oral dryness) OR (hyposalivation) OR (asialia) OR (dryness of the mouth) OR (“xerostomia”[MeSH Terms]) OR (mouth dryness) OR (dry mouth). For this study MeSH terms were used to increase our search. The initial search was combined with the following terms: AND (“acupuncture”[MeSH Terms]) OR (“acupuncture therapy”[MeSH Terms]) OR (acupuncture) OR (“moxibustion”[MeSH Terms]) OR (moxibustion) OR (electroacupuncture) OR (electro acupuncture) OR (“acupuncture, ear”[MeSH Terms]) OR (ear acupuncture) OR (ear/electro acupuncture). For the search of Web of science exactly the same terms were used, but without MeSH terms. Manual search was carried out to enrol other potentially relevant articles, which could not be found with the electronic search. Therefore, the reference lists of the included articles were checked for further possible trials.

### Selection criteria

Two authors (Z.A. and H.B.) independently searched for articles and independently examined the title and abstract of all records identified. The authors assessed each of these articles to determine which met the inclusion criteria for this review. For all articles that seemed to meet the inclusion criteria, a full text version of was retrieved. The inclusion criteria used for the present study were:

- Articles in English or Dutch
- Randomized controlled trials (RCT)
- Patients with dry mouth symptoms (xerostomia) or hyposalivation due to any cause
- Dentate and/or edentulous patients
- Studies using invasive acupuncture (acupuncture with needle penetration of the skin)
- Studies using one or more of the following parameters for oral dryness were eligible: salivary flow rate (unstimulated whole saliva (UWS) or stimulated whole saliva (SWS)), salivary gland scintigraphy, functional magnetic resonance imaging of salivary glands, or subjective parameters (Visual Analogue Scale (VAS), Xerostomia Questionaire, Quality of life, duration of effectiveness, patient satisfaction with treatment) [23, 24].

Excluded were systematic reviews (plus meta-analysis), cohort studies, case-control studies, in vitro studies, case reports/series, letters to the editor and studies using non-invasive acupuncture.

Any disagreement between the two authors was resolved by discussion.

### Quality assessment

The methodological quality of the included randomized controlled trials (RCT) was assessed using the Cochrane Collaboration’s tool described in Handbook version 5.1.0 [25]. Table 1 shows the potential biases assessed in the present study. The same authors who conducted the search for the articles assessed the quality of each RCT. These authors independently assessed each RCT for the risk of bias. Differences in rating between authors were resolved by discussion. Studies with high risk in one or more domains were rated as high risk of bias (plausible bias that seriously weakens confidence in the results). Studies were only rated as low risk of bias (plausible bias unlikely to seriously alter the results) when the study met the criteria in all domains. Studies were rated as unclear risk of bias (plausible bias that raises some doubt about the results) if there was unclear risk or if there was no clear description of the implemented method in one or more domains.

**Table 1:** Potential risks of bias, assessed in the present study [25]

Random sequence generation	selection bias
Allocation concealment	selection bias
Blinding of participants	performance bias
Blinding of practitioners	performance bias
Blinding of outcome assessment	detection bias
Incomplete outcome data	attrition bias
Selective reporting	reporting bias
Other bias	

## Results

**Fig. 1:**
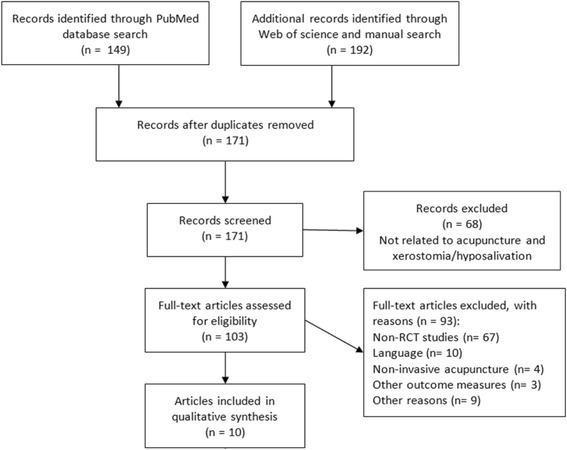
Prisma flow diagram of the systematic review process

The initial search of Pubmed and Web of Science, and the subsequent manual search resulted in a total of 341 possible articles (Fig. 1). After removing duplicates, a total of 171 articles were initially identified. Based on the titles and abstracts of these publications, 68 articles were discarded by the two reviewers as being not related to this systematic review, because they did not discuss acupuncture and xerostomia/hyposalivation. One hundred and three references were retrieved in full text. Of these references 93 articles were excluded for several different reasons. The major reason for exclusion was that studies did not have a RCT design. The language of some articles (Swedish, Russian, French, Spanish, German, and Czech) was another reason to exclude these articles. In addition, several articles describing non-invasive acupuncture procedures (like acupuncture-like transcutaneous nerve stimulation, or laser acupuncture) were also excluded. The use of other outcome measures like blood flux of the skin, and the salivary concentration of peptides were another reason to exclude articles. Finally, nine publications were excluded because of several other reasons: congress abstracts of included articles (*n* = 4), articles not discussing a relation between acupuncture and xerostomia/hyposalivation (*n* = 4), and a research using healthy volunteers (*n* = 1). After removing all excluded publications, 10 studies met the inclusion criteria for the systematic review.

### Included studies

#### Characteristic of the trial design and settings

Ten RCTs met the inclusion criteria and were included in this systematic review. Table 2 summarizes important characteristics of the included articles. All studies had a parallel group trial with two arms comparing an experimental arm (acupuncture) with a control arm. The experimental groups consisted of the following acupuncture methods: auriculotherapy [26], acupuncture according to traditional Chinese medicine [27, 28], acupuncture according to traditional Chinese and orthodox Western medicine [29], acupoints mainly in the regions of the parotid, submandibular and labial glands [30], acupuncture according to traditional Chinese medicine and biomedicine [31], real acupuncture (using different body points) [32, 33], acupuncture using standard and customized anatomic points [34], acupoints in the bilateral ears, index finger and an additional facial point [35]. For the control groups, the studies used also different methods. The following control groups have been used: placebo auriculotherapy, placebo acupuncture (superficial needling) [27, 28], no therapeutic modality at all [29, 30], sham acupuncture (non-acupoints 2 cm away from the real acupoints) [33], standard care group (oral hygiene) [31], sham acupuncture (non-penetrating needle device) [32], usual care (no specific treatment) [34], oral care sessions (lifestyle and dietary advices) [35]. Only one study used a cross-over design [35], with crossover 4 weeks after the end of the first intervention (acupuncture or oral care sessions). One study had a mixed design of a RCT and a cohort study [30]. Patients were first randomized between acupuncture and the control group. After finishing a 10 weeks’ period the control group were treated with acupuncture as well. Three trials studied the preventive effect of acupuncture in minimizing/preventing xerostomia among cancer patients before and during radiation therapy [29, 31, 32]. Three trials were conducted in Sweden [27, 28, 30], two in China [31, 32], one in France [26], one in Brazil [29], one in South Korea [33], one in the USA [34], and one in the UK [35]. All studies were single centre studies, except the study in the UK, in which seven oncology centres participated [35].

**Table 2:** This table shows the characteristics of the included randomised controled trials

Articles	Year	Country	Type of patients	Total of patients	Type of acupuncture	Type of control group	Studying preventive acupuncture effect	Outcome measures
Alimi [26]	2012	France	Receiving radiotherapy for head and neck cancer	60	Auriculotherapy acupuncture	Sham/placebo acupuncture	No	VAS scores
Blom [27]	1992	Sweden	Variety of patients	21	Traditional Chinese medicine acupuncture	Sham/placebo acupuncture	No	SFR and changes in subjective symptoms
Blom [28]	1996	Sweden	Receiving radiotherapy for head and neck cancer	41	Traditional Chinese medicine acupuncture	Sham/placebo acupuncture	No	SFR and changes in subjective symptoms
Braga [29]	2011	Brazil	Receiving radiotherapy for head and neck cancer	24	Traditional Chinese and orthodox Western medicine acupuncture	Oral hygiene/usual care	Yes	SFR and VAS scores
Cho [33]	2008	South Korea	Receiving radiotherapy for head and neck cancer	12	real acupuncture	Sham/placebo acupuncture	No	SFR and XQ scores
List [30]	1998	Sweden	Sjögren’s syndrome patients	21	Parotid, submandibular and labial glands acupuncture	Oral hygiene/usual care	No	SFR and VAS scores
Meng [31]	2012	China	Receiving radiotherapy for head and neck cancer	86	Traditional Chinese medicine and biomedicine acupuncture	Oral hygiene/usual care	Yes	SFR and XQ scores
Meng [32]	2012	China	Receiving radiotherapy for head and neck cancer	23	Real acupuncture	Sham/placebo acupuncture	Yes	SFR and XQ scores
Pfister [34]	2010	USA	Receiving neck dissection and radiotherapy for cancer	70	Standard and customized anatomic points acupuncture	Oral hygiene/usual care	No	Xerostomia Inventory
Simcock [35]	2012	UK	Receiving radiotherapy for head and neck cancer	145	Bilateral ears, index finger and an additional facial point acupuncture	Oral care sessions	No	SFR. Quality of Life Questionnaire and the Head and Neck subscale

*SFR* salivary flow rate, *UK* United Kingdom, *USA* United States of America, *VAS* visual analogue scale, *XQ* xerostomia questionnaire

Six studies were funded solely by research grants from publicly funded bodies [28, 30–33, 35]. The remaining four trials did not state the sources of funding for the studies [26, 27, 29, 34].

#### Characteristics of the participants

A total of 503 participants took part in the ten trials with a mean of 62 participants per trial and a range of 12 to 175. All participants were adults with different causes of xerostomia. One study included only patients with primary Sjögren’s syndrome [30]. In seven trials the cause of xerostomia was radiotherapy for head and neck cancer [26, 28, 29, 31–33, 35], sometimes in combination with chemotherapy. Furthermore, one study included patients who had undergone neck dissection and radiotherapy for cancer [34]. In the remaining study participants suffered from a variety of causes of xerostomia, including primary and secondary Sjögren’s syndrome, radiotherapy and hypothyroidism [27].

#### Characteristics of the intervention

All the trials evaluated acupuncture. Only one study used both manual stimulation and electrical stimulation of two acupuncture points [30]. Five trials compared acupuncture to sham/placebo acupuncture [26–28, 32, 33]. Four trials compared acupuncture to oral hygiene/usual care [29–31, 34]. The patients in the control group of oral hygiene/usual care did not receive any therapeutic modality. Only one clinical trial used oral care sessions as control group [35]. During these sessions dietary and lifestyle advices to improve xerostomia where given to patients. The duration of acupuncture treatment varied between the clinical trials. The acupuncture treatment lasted for 6 weeks in most of the included studies [27, 28, 32, 33], separated in one study [28] by a 2 weeks resting period. Other studies used 4, 7, 8 (two studies) or 10 weeks of acupuncture treatment [26, 30, 31, 34, 35]. In one study the experimental period varied between 8 and 10 weeks [29].

#### Characteristics of the outcomes

Six studies used a combination of objective and subjective outcome measures. Two studies used the salivary flow rate (unstimulated as well as stimulated) in combination with a VAS xerostomia questionnaire to measure the effect of acupuncture [29, 30]. Three studies [31–33] used a combination of salivary flow rates and Xerostomia Questionnaire (XQ) to assess dry mouth. Finally, one study measured the unstimulated and stimulated flow rates in combination with the European Organisation for Research and Treatment of Cancer Quality of Life Questionnaire and the Head and Neck subscale as subjective outcome measures [35].

Four studies used either objective or subjective outcome measures [26–28, 34]. Alimi et al. [26] used a VAS for dry mouth, while Pfister et al. [34] used the Xerostomia Inventory, a validated questionnaire. In the two studies by Blom and co-workers [27, 28], the unstimulated and stimulated salivary flow rates were reported, as well as changes in subjective symptoms and changes in the medication during or after the acupuncture procedure. *Risk of bias in included studies (*Table 3).

**Table 3:** The risk of bias in all the included studies (top-down) according to Cochrane Collaboration’s biases tool (from left to right). The plus sign indicating low risk of bias whereas the minus sign indicates high risk of bias. The question mark indicates an unknown risk of bias

	Random sequence generation (selection bias)	Allocation concealment (selection bias)	Blinding of participants (performance bias)	Blinding of practitioners (performance bias)	Blinding of outcome assessment (detection bias)	Incomplete outcome data (attrition bias)	Selective reporting (reporting bias)	Other bias
Alimi 2012 [26]	+	+	+	–	+	+	–	+
Blom 1992 [27]	+	–	+	–	+	–	+	–
Blom 1996 [28]	+	–	+	–	+	–	+	–
Braga 2011 [29]	–	–	–	–	–	+	+	–
Cho 2008 [33]	+	+	+	–	+/?	+	+	–
List 1998 [30]	+	–	–	–	−/+	–	–	–
Meng 2012 [31]	+	+	–	–	−/?	–	+	+
Meng 2012 [32]	+	+	+	–	+/?	–	+	+
Pfister 2010 [34]	+	+	–	–	–	+	+	–
Simcock 2013 [35]	+	+	–	–	–	–	+	+

+ = low risk of bias

- = high risk of bias

? = unknown risk of bias

#### Allocation

With the exception of one study [29] all studies have adequate sequence generation (Table 3). Four did not describe an adequate allocation of concealment [27–30] and therefore these trials were assessed as high risk of selection bias.

#### Blinding

Blinding of participants to the allocated treatment by the use of placebo acupuncture (sham acupuncture) was done in five of the included studies [26–28, 32, 33]. These trials were assessed as low risk of performance bias. The other five studies were assessed as high risk of performance bias, because the participants were not blinded to the allocated treatment.

Blinding of the practitioner was not observed in any study. For this reason, all the included studies had high risk for this part of performance bias.

The outcome assessors (the patient or the examiner) were blinded with regard to the treatment in three trials [26–28] and these trials were assessed as low risk of detection bias. Three other studies did not blind the outcome assessors and were judged as high risk of detection bias [29, 34, 35]. The remaining studies did not report clear information concerning the blinding of the outcome assessors.

The study of Cho et al. [33] had a low risk and an unknown risk of detection bias. In this study, the patients were blinded to the allocated treatment, so when they administer the self-reported XQ-questionnaire, no risk of bias would be expected, as the patients did not know which treatment they received. However, the publication did not mention whether the outcome assessor was also blinded to the allocated treatment. Therefore, the risk of detection bias for this part of the study is unclear. The study of List et al. [30] had a high and a low risk of detection bias. The participants were not blinded with regard to their treatment. Because the participants were aware of their treatment this results in a potential detection bias for the VAS questionnaire. The salivary secretion rate was measured by one person while the acupuncture treatment was performed by another person. Therefore, this part of the study was assessed as low risk of detection bias. Meng et al. [31] had a high risk and an unknown risk of detection bias. In this study the patients were not blinded to the allocated treatment, resulting in a potential detection bias for the Xerostomia Questionnaire, because the participants were aware of their treatment. It was unclear from the publication who measured the salivary secretion rate and subsequently the risk of detection bias for that part of the study was assessed as unknown. Meng et al. [32] had a low and unknown risk of detection bias. In this study, the participants were blinded to the allocated treatments resulting in a low risk of detection bias for the Xerostomia Questionnaire. However, in this study it was unclear who measured the salivary secretion rate, the reason why for that part of the study the risk of detection bias was assessed as unknown.

#### Incomplete outcome data

Four trials [26, 29, 33, 34] were assessed as low risk of attrition bias, because no drop out was reported or the intention to treat principle had not been used to evaluate the outcome data. The other six trials were assessed as high risk of attrition bias: Blom et al. [27] did not mention why some patients did not complete the treatment and some data with regard to the salivary flow rate are missing without any explanation. Four studies [28, 30, 31, 35] had a high risk of attrition bias because there was selective drop out in either the experimental group or the control group. Although Meng et al. [32] had similar numbers of dropouts in both arms of the trial, the initial dropout rate was high for the participants in both groups. Hence this article was assessed as high risk of attrition bias.

#### Selective reporting

Eight publications articles were assessed as low risk of reporting bias. Alimi et al. [26] reported that they used VAS to measure dry mouth and pain. However, no data were reported about pain. On the other hand, they reported results of mouth moistening (in liters!) and effectiveness of the acupuncture needles over time. For these reasons this publication was assessed as high risk of reporting bias. List et al. [30] reported in their methods that labial salivary gland biopsy data would be collected. As these data were not reported, this article is also assessed as high risk of reporting bias.

#### Other sources of bias

Four of the included trials [26, 31, 32, 35] were assessed as low risk of other bias. The remaining six trials were assessed as high risk of other bias. The most common source for bias was inconsistent acupuncture protocol. Blom et al., Pfister et al. [27, 28, 34] did not use the same number of acupuncture points in all patients. The number of acupuncture points used depended on the patients’ particular complaints and their general health. On the other hand, Braga et al. [29] gave the patients different numbers of acupuncture sessions (16–20 sessions). Another point for high risk was inconsistent penetration depth of the acupuncture needles. Blom et al., Braga et al., Pfister et al. [27–29, 34] did not use a similar depth for the acupuncture needles. Cho et al. [33] did not have a standardized depth (less than 0,5 cm) of the acupuncture needles for the control group (sham acupuncture). In Blom et al. [27, 28] the distance between the classical acupuncture point and the placebo acupuncture point was not standardized but varied between 1 and 2 cm. Another problem in some acupuncture protocols was electric stimulation of some acupuncture points. In the publication by List and co-workers [30] one group got electrical stimulation of 2 acupuncture points while another group only received manual stimulation of all the acupuncture points.

A concerning point about the trial of Pfister and co-workers [34] is the study participants would not return to complete the final assessments, a fifth acupuncture treatment was added to enhance compliance.

The use of block randomization was another point of bias. In the study of Cho et al. [33] this resulted in an uneven distribution of subjects with regard to disease characteristics. This unbalanced grouping was the result of the use of block randomization.

#### Overall risk of bias

All of the included trials in this review had at least one domain where risk of bias was high (see Table 3). Consequently, all the trials were assessed as high risk of bias. The lowest risk of bias was observed for the study by Alimi et al. [26], the highest for the study by List and co-workers [30].

### Effects of the intervention

In this section, acupuncture compared to other interventions will be discussed. These interventions include sham/placebo acupuncture, regular (oral) care and other treatments. The outcomes measures are unstimulated saliva secretion rate, stimulated saliva secretion rate and subjective outcome measures.

#### Acupuncture versus sham/placebo acupuncture

##### Unstimulated saliva

Four trials with high risk of bias reported data for the unstimulated secretion rate. Meng et al. [32] investigated the effect of acupuncture before and during treatment with radiotherapy. During a period of 11 weeks the unstimulated salivary flow rate decreased significantly over time. After 3 weeks there was no difference between acupuncture and sham procedure. After 6 weeks real acupuncture-treated patients had an approximately 50% higher salivary flow rate compared to sham acupuncture patients, but this difference was not statistically significant.

The three other studies [27, 28, 33] investigated the effect of acupuncture in patients that had been treated with radiotherapy in the past or in patients suffering from severe xerostomia associated with a systemic disease. Cho et al. [33] measured the salivary flow rate for a period of 6 weeks. Blom et al. [27, 28] measured the flow rates for a longer period (12 months).

Using acupuncture, Cho et al. [33] noticed that after 3 weeks the unstimulated flow rate had increased by 55% compared to baseline and after 6 weeks with 75%. However, acupuncture only significantly improved saliva secretion at 6 weeks. In the sham treated population, after 3 and 6 weeks the unstimulated salivary flow rate was 2 and 15% higher, respectively, than the base line. Although these values suggest that patients benefit more from acupuncture, the differences between the two experimental groups were not significant at any time point.

Blom et al. [27] reported a significant increase of the unstimulated flow rate versus baseline in the acupuncture group at all time points from 7 to 64 weeks. In the placebo group a significant increase versus baseline was observed only at 16 weeks. After 28 weeks and 40 weeks the difference in salivary flow rate was significant in favour of acupuncture.

In the trial of Blom et al. [28], both acupuncture and placebo showed a significant effect on the unstimulated salivary flow rate at all the time points compared to baseline, except for 6 months in the placebo group, which did not reach any statistical significance. When comparing acupuncture and placebo, the differences were not statistically significant.

##### Stimulated saliva

In the study of Meng et al. [32] on the effect of acupuncture before and during radiotherapy, a significant decrease of the stimulated flow rate over time was observed in both the treatment and control group. At baseline, week 3 and week 6, the average flow rate of the acupuncture group was 24, 0 and 36%, respectively, higher than that of the sham-treated group. There was no statistical significant difference, however, at any time point. At the end (week 11) there was only 8% difference between acupuncture and sham procedure.

Cho et al. [33] noticed that after 3 weeks the stimulated flow rate of patients with radiation-induced xerostomia increased by 3% compared to baseline using acupuncture and after 6 weeks the flow rate had increased with 20% compared to baseline. The sham procedure induced a decrease in salivary flow rate: at week 3 and 6 the flow rate had decreased respectively 9 and 5% compared to baseline. However, the difference between the two treatments was not significant.

Blom et al. [27] reported an increase of the stimulated flow rate both in the acupuncture and in the placebo group. When comparing acupuncture and placebo, significant differences in favour of acupuncture were seen at all time points, except 16 weeks. The acupuncture group showed significant differences at all time points versus baseline. In contrast, in the placebo group no significant differences were found.

The other study of Blom et al. [28] included only patients who had all or some of their salivary glands irradiated. In this study a significant increase was observed for the stimulated flow rate for most of the time points versus the baseline flow rates in both the acupuncture and the placebo group. Only at week 8 and 12 the flow rate of the placebo group did not differ significantly from the baseline value. Although acupuncture seemed to be better compared to placebo, no significant effect was seen between the two groups at any time point.

##### Perceived dry mouth

Alimi et al. [26] measured variations in the intensity of dry mouth using a VAS. Acupuncture induced a significant 66% improvement of the VAS score compared to 4% for the sham procedure.

Meng et al. [32] quantified the dry mouth feeling using the Xerostomia Questionnaire (XQ). The sham group had after 3 and 6 weeks significantly higher XQ scores than the acupuncture group, respectively 37 and 43% in favour of acupuncture. The greatest difference was seen at week 11: 56%.

Cho et al. [33] also used the Xerostomia Questionnaire. In both the treatment and the placebo group the subjective dry-mouth complaints improved compared to the baseline. However, no statistical difference in XQ score between the two groups was found at any time point.

Blom et al. [27, 28] did not use a questionnaire to quantify the dry mouth feeling but reported any changes of subjective symptoms during or after the treatment. Blom et al. [27] noted that in the experimental group two patients reported that they had less viscous saliva while in the control group none of the patients reported such change. Blom et al. [28] reported that many patients (number not reported) experienced a decrease of mucus secretion and a more fluid saliva. Some patients also reported improved taste, diminished pain of the tongue and less hoarseness. Those changes were somewhat slower and weaker in the control group, but these changes were not quantified.

#### Acupuncture versus regular (oral) care

##### Unstimulated saliva

Three trials with high risk of bias reported data on the effect of acupuncture on unstimulated saliva secretion. Braga et al. [31] and Meng et al. [29] investigated the effect of acupuncture before and during radiotherapy for a period of 8 to 10 weeks, and for a period of 6 month, respectively. In the study of Meng et al. [31] the unstimulated salivary flow rate decreased over time during radiotherapy. The acupuncture group had significantly higher flow rates from week 3 up to week 11 when compared to standard oral hygiene, with the greatest group difference at week 7 (group difference of 0.06 g/min). However, after 6 months, the difference between the two groups was no longer statistically significant.

Braga et al. [29], who did not monitor the salivary flow rate over time reported a significant difference between the acupuncture group and the control group of 425% in favour of acupuncture.

The follow up period in the trial of List et al. [30] in patients with Sjögren’s syndrome was 10–20 weeks. No significant effects versus baseline were found for both the acupuncture and the control group, and no significant differences between the two groups were observed.

##### Stimulated saliva

The trial of Meng et al. [31] showed that patients receiving radiotherapy treated with acupuncture had significantly higher flow rates compared to standard oral hygiene from week 4 that remained through till 6 months. The greatest group differences were observed at week 7 (group difference of 0.112 mL/min).

Also Braga et al. [29] found significant changes between the acupuncture–treated and the control group. After radiotherapy the differences in stimulated flow rate between these groups were 308% in favour of acupuncture.

In the trial of List et al. [30] the median of the salivary flow rate increased with 100% in patients with Sjögren’s syndrome after acupuncture treatment compared with baseline. However, no statistical significant differences between the acupuncture group and the control group were observed.

##### Preceived dry mouth

Pfister et al. [34] measured the dry mouth symptoms using the Xerostomia Inventory (lower scores indicate a better outcome). The follow up period lasted for 4 weeks. After 4 weeks, there was a decrease of 12% for the XI scores in the acupuncture group versus baseline. For the control group (usual care) a decrease of 2% was seen after 4 weeks. The differences between these two groups were statistically significant.

Meng et al. [31] measured the dry mouth feeling using the Xerostomia Questionnaire. Starting in week 3, the control group had significantly higher XQ scores than the acupuncture group. This difference lasted for 6 months. The absolute differences between the groups increased over time with the greatest difference observed at week 7 (group difference of 10.3). After 7 weeks the difference between the two groups was still significant and the group difference was comparable with week 7.

Braga et al. [29] used a VAS to score the following dry mouth symptoms: ‘difficulty in speaking’, ‘difficulty of swallowing’, ‘quantity of saliva in the mouth’ and ‘dryness of the mouth’. The VAS scores of these items revealed statistically significant differences between the acupuncture group and the control group. The score for the item ‘difficulty in speaking’ was in the control group 57% higher than in the treated group. The scores for ‘difficulty swallowing’ and ‘the dryness of the mouth’ were 57 and 47%, higher in the treatment group compared to the acupuncture group. The largest difference between the two groups was found for the item ‘quantity of saliva in the mouth’, which scored in the acupuncture group 239% higher than in the control group. This indicates that acupuncture treatment had a positive effect on the perceived quantity of saliva in the mouth. List et al. [30] evaluated dry mouth symptoms in the experimental and control groups with VAS items exploring discomfort caused by dryness of the mouth and the eye, and by a burning sensation of the mouth. The scores of eye dryness will not be included in this review. In the test group the perceived mouth dryness decreased significantly versus baseline (the median decreased with 24%), whereas in the control group no significant differences were found (the median increased with 8%). When comparing the acupuncture group with the control group, no significant differences were found after acupuncture treatment (a difference of 19% of the median between the two groups). No effect of acupuncture treatment on the sensation of burning mouth was found. After 10 weeks the control group also received acupuncture. Subjective evaluation of discomfort caused by mouth dryness, eye dryness and burning sensation in the mouth was performed. In addition, reduction in speech, chewing ability and the effect of dry mouth on daily activities were evaluated. Acupuncture treatment had no effect on any of these subjective outcomes.

#### Acupuncture versus other treatments

##### Unstimulated and stimulated saliva

The trial of Simcock et al. [35] was the only trial (with high risk of bias), which compared acupuncture with another treatment. In this study acupuncture was compared with oral care sessions. During oral care sessions lifestyle and dietary advice were given to patients. Additionally, this was the only study which had a cross-over design. Group one started with oral care sessions first and after a wash out period of 4 weeks they got the acupuncture treatment. Group two started with the reverse order of interventions.

There were no significant changes in either unstimulated saliva or stimulated saliva over time or by intervention.

##### Preceived dry mouth

In the same study [35] subjective dry mouth symptoms were evaluated using the Quality of Life Questionnaire and the Head and Neck subscale. Dry mouth symptoms were explored with questions about, “dry mouth”, “sticky saliva”, “need to sip liquids to relieve a dry mouth”, “need to sip to swallow food”, “dry lips” and “waking up at night because of need to drink”. For every item, the odds ratio of reduced symptoms following acupuncture versus oral care were given. Significant odds ratios were found for dry mouth symptoms (2.01), sticky saliva (1.67) the need to sip to swallow food (2.08) and waking up at night to drink (1.71). No significant effect of acupuncture was seen on the odds ratio for dry lips (1.65) and sipping of liquids to relieve a dry mouth (1.59).

## Discussion

### Summary of main results

The ten studies included in this review were classified into three categories based on the comparison groups: sham acupuncture, regular oral care or other treatment. The quality for all the main outcomes has been assessed as low. Although some publications suggest a positive effect of acupuncture on either salivary flow rate or subjective dry mouth feeling, the studies are inconclusive about the potential effects of acupuncture.

### Quality of evidence

None of the trials included in this review are at low risk of bias. There was a huge difference in number of participants per trial (varying from 12 to 145 participants). Only one study [34] reported a power analysis. As most other studies did not report sample size calculations these studies are likely to lack statistical power to detect differences between both arms of the trial. This can result in a type II error, the erroneous conclusion of no effect between treatments arms [36, 37]. In trials where several primary outcome measures are studied, such as salivary flow rate as well as xerostomia symptoms, the power needs to be set at a higher level (> 90%) for each endpoint [38].

In one study [29] randomization and concealment of allocation was not performed. This can introduce selection bias with significant effects on the results of a study. It has been reported that lack of random allocation with adequate concealment can have effects as large or larger than the expected effects of the intervention [39].

Blinding of the participants, practitioners and/or outcome assessor was not done in most of the studies. If bias is introduced during a trial because of differential treatment of groups or biased assessment of outcomes, no analytical techniques can correct for this limitation. Subsequently the results from unblinded trials should be interpreted with caution [40]. In a systematic review of 250 RCTs identified from 33 meta-analyses, researchers observed a significant difference in the size of the estimated treatment effect between trials that reported “double-blinding” compared with those that did not (*p* = 0.01) [41]. Another study of Jüni et al. showed that the results for double blinded studies were more heterogeneous [42]. A meta-analysis of four empirical studies relating key aspects of methodological quality to the effect estimates of controlled trials, revealed that that estimates were on average moderately biased in open trials (odds ratio of 0.83 and 0.88). In contrast, of the two smaller studies, one did not report an effect (odds ratio 1.11), whereas the other concluded that lack of double blinding introduced substantial bias (odds radio 0.56) The combined odds ratio for bias associated with the lack of double blinding is 0.86, further supporting the importance of blinding in a RCT [42]. On the other hand, Balk and co-workers in their meta-analyses of RCTs did not find any consistent associations between double blinding and the magnitude of the treatment effect [43].

Although sham acupuncture seems to be a good placebo procedure to blind the participants, several authors of included studies suggests that the sham procedure itself can have a beneficial effect on dry mouth. According to the philosophy of traditional Chinese medicine, there could be some positive effect of an acupuncture needle even when the needle is not placed accurately [27]. Blom and co-workers expressed that sham acupuncture with superficial needling could not be considered a placebo procedure and should be considered a different type of acupuncture treatment with less sensoric stimulation [28]. Another study of Vincent et al. shows that undifferentiated peripheral stimulation (needling) may have certain therapeutic effects, for example in pain reduction [44]. If sham acupuncture also has effects, its use as a comparison condition with true (point-specific) acupuncture may impose an unrealistic burden of proof upon the latter. This has important implications for the setup of such studies since very high subject numbers are required in order to be able to reveal a small additional effect of true acupuncture over sham treatment [44]. Thomas et al. conducted a controlled study investigating the effect of acupuncture on chronic nociceptive low back pain. They also conclude that ‘placebo’ acupuncture is a contradiction, since any sensory stimulus provokes a physiological response and thus cannot be inert [45]. This means that consideration should be given to designing a different control intervention.

Blinding of the practitioners was not done in any of the included studies, so performance bias can have influenced the effects in all included studies. Some of the studies [29–31, 34, 35] also did not blind the outcome assessors. Blinding of outcome assessors can be especially important for assessment of subjective outcomes, such as dry mouth symptoms. When no blinding of outcome assessor is done, detection bias may affect the results. Hróbjartsson conducted a systematic review of randomized clinical trials with both blinded and non-blinded assessment of the same measurement scale outcome. They included 16 trials with subjective outcomes. Their study provides empirical evidence for observer bias in randomized clinical trials with subjective measurement scale outcomes. Non-blinded assessors exaggerated the pooled effect size by 68% (95% CI 14% to 230%) [46]. Another study reached a similar conclusion for binary subjective outcomes. Non-blinded assessors of subjective outcomes generated substantially biased effect estimates in randomised clinical trials, exaggerating odds ratios by 36% [47]. The exaggerating of odds ratios in studies with patients being outcome assessors, as in the studies of our review, is unknown, but might be comparable to that of physicians.

Withdrawals from the study lead to incomplete outcome data, which can cause attrition bias. Attrition bias can affect the strength of a trial’s findings [48]. Of the studies in this review, Meng et al. [31] showed the highest dropout rate (22%), without disclosing how these were distributed over the two groups. Uneven distribution of dropouts over the different arms of a study population, potentially invalidates the conclusions of a study. Another factor which may negatively impact the reliability of an RCT is the lack of a standard acupuncture protocol. This is a problem potentially affecting the quality of the majority of the included studies. Only four studies [26, 31, 32, 35] used a standard acupuncture protocol for treatment of the participants. Of these four studies only one [31] showed a significant effect of acupuncture on the salivary flow rate. When looking to the other six studies using a non-standardized protocol, two studies [27, 29] show a significant effect on salivary flow rate.

### Overall completeness and applicability of evidence

An important consideration is the variation between the participants in these trials. The nature and extent of the salivary gland disease is likely to vary between participants with resultant variations in residual gland function, disease history and prognosis among the participants. Only trials that included patients treated with radiotherapy and trials with a heterogeneous group of patients show a significant effect of acupuncture. A trial that included a heterogeneous group of participants [27] did report a significant effect on the salivary flow rate, but it is unclear whether subgroups of patients in this study - which included radiotherapy patients, Sjögren patients and patients suffering from hypothyroidism - are responsible for this effect. The trial that included patient with Sjögren’s disease only, did not report a significant effect of acupuncture. Notably, studies using other forms of acupuncture did report effects in Sjögren’s patients. For instance, in a randomized placebo controlled study a positive effect of laser acupuncture was reported on the salivary flow rate in patients with Sjögren’s syndrome compared to that in the control group (sham treated laser acupuncture) [49].

Blom et al. measured the effect of acupuncture on local blood flux in patients suffering from Sjögren’s syndrome [16]. Patients with Sjögren’s syndrome showed an increase in the peripheral vascular flux, which may be an important factor in relief of xerostomia [16].

A factor which complicates the comparison of the outcomes of different studies in cancer patients are differences in treatment modality, e.g. type of radiotherapy, radiation dose, or the type of chemotherapy. A source of variation is the type of radiotherapy that patients received who participated in these studies. In all but three studies [32, 34, 35] the radiotherapy technique used was not specified precisely. Knowledge of the type of radiotherapy patients underwent is important, because late toxicities, including xerostomia and Quality of Life are dependent on the treatment modality [50]. Meng et al. [32] only included patients treated with Intensity-Modulated Radiation Therapy (IMRT). This radiation technique minimizes the dose to surrounding normal tissue [51]. Duarte et al. showed that IMRT patients exhibited significantly less xerostomia compared with those treated with conventional radiation therapy [52]. This may explain why this study did not find any effect of acupuncture on the salivary flow rate.

Another potential source of variation between studies is the impact of treatment modality. Only four studies [29, 32, 33, 35] clearly mentioned which treatment modality (chemoradiation or only radiation) the patients received. Treatment modality is important because it effects the acute (as example mucositis and dermatitis) and late toxicity (as example dysphagia and xerostomia). The incidences of late toxicity side effects were significantly increased in patients treated by chemoradiation, compared to radiation alone [53]. On multivariate analysis, chemotherapy and radiation technique showed a significant correlation with the incidence of late toxicity [53].

### Potential biases in the review process

We conducted a broad search of two different databases and placed restrictions on the language of publication when searching the electronic databases or reviewing reference lists of the included studies. Subsequently it is likely that other studies, published in Chinese journals, may not have been identified for this review. Morrison et al. found no evidence of a systematic bias in conventional medicine studies from the use of language restrictions in systematic review-based meta-analyses [54]. Pham et al. also found the same for conventional medicine. However, the results of systematic reviews of complementary and alternative medicine do substantially alter when language restrictions are used [55, 56]. However, a team of authors based in China identified the same four RCTs that we included in this review, although they searched both English and Chinese databases [23]. This makes it unlikely that Chinese publications on acupuncture would have altered the conclusions of the present systematic review.

We decided to include cross-over studies in this review. A systematic review about non-pharmacological interventions for the management of dry mouth excluded cross-over studies [24], because the beneficial effects of acupuncture might last for weeks or months after the end of the treatment. A non-RCT retrospective study of Blom et al. confirms these results [4]. This non-RCT study shows that acupuncture treatment results in statistically significant improvements in salivary flow rate in patients with xerostomia up to 6 months. It even suggests that additional acupuncture therapy can maintain this improvement in salivary flow rate for up to 3 years. This means that inclusion of cross-over is an important potential limitation of the present review, as the washout period (4 weeks) in the included cross-over study of Simcock et al. [35] was relatively short.

### Agreement and disagreement with other studies reviews

Several other studies have investigated the effect of acupuncture on healthy subjects. Dawidson et al. investigated the influence of acupuncture on the salivary flow rates of healthy students or dentists [57]. Unstimulated salivary flow rate showed a significant increase both during and after acupuncture stimulation compared to baseline levels, while stimulated salivary flow rates did not show any significant changes [57]. Deng et al. conducted a randomized, sham acupuncture controlled, subject blinded trial [58]. They included 20 healthy volunteers who received true and sham acupuncture in random order. True acupuncture led to a significantly higher saliva production compared to sham acupuncture [58]. These studies suggest that acupuncture also can have an effect on the saliva secretion of healthy subjects.

Some patients with dry mouth symptoms might benefit from acupuncture, but in absence of good evidence of their effectiveness their clinical relevance is questionable. Especially the costs of acupuncture make wide use less favourable. Based on current practice rates in the US, the cost of acupuncture are estimated at $400–$600 per treatment course [59]. Taken together, this does not seem to justify the use of acupuncture outside clinical trial setting at this moment.

## Conclusions

All the included studies had a high risk of bias affecting the evidence of the studies. There is some evidence that acupuncture can increase salivary flow rate and/or alleviate dry mouth symptoms in patients following radiotherapy or in a heterogeneous group of patients. These results need to be interpreted with caution because of the high risk of bias in the included studies (low quality evidence). Overall there is insufficient evidence to determine the effect of acupuncture on dry mouth or hyposalivation symptoms. Acupuncture did not show any significant effect on the saliva production or dry mouth symptoms in patients with Sjögren’s syndrome.

Further well-designed, double blinded trials with sufficient number of participants are required to determine the potential benefit of acupuncture. Sample size calculations should be done before before initiating the study. Trials should be designed and conducted according to SPIRIT 2013 statement guidelines and reported according to the CONSORT 2010 statement guidelines. These trials should not only include salivary secretion rates and validated xerostomia questionnaires, but also other important outcomes like dry mouth symptoms, quality of life, duration of effectiveness and patient’s satisfaction with the intervention. During these clinical studies, acupuncture could be compared with other promising potential treatments for hyposalivation e.g. low-laser therapy [60].

## Abbreviations

ACTA: Academic Centre for Dentistry Amsterdam
CAM: Complementary and Alternative Medicine
CI: Confidence interval
CONSORT: Consolidated Standards of Reporting Trials
HCV: Hepatitis C Virus
HIV: Human Immunodeficiency Virus
IMRT: Intensity-Modulated Radiation Therapy
MeSH: Medical Subject Headings
RCT: Randomized controlled trials
SPIRIT: Standard Protocol Items: Recommendations for Interventional Trials
SWS: Stimulated Whole Saliva
UK: United Kingdom
US: United States
USA: United States of America
UWS: Unstimulated whole saliva
VAS: Visual Analogue Scale
XI: Xerostomia Inventory
XQ: Xerostomia Questionnaire
